# Effect of Selective Encapsulation of Hydroxypropyl-β-cyclodextrin on Components and Antibacterial Properties of Star Anise Essential Oil

**DOI:** 10.3390/molecules23051126

**Published:** 2018-05-09

**Authors:** Guangjie Zhang, Chao Yuan, Yonghai Sun

**Affiliations:** 1 College of Food Science and Engineering, Jilin University, No. 5333, Xi’an Road, Changchun 130062, China; zhangguangjie000@163.com; 2 School of Biotechnology and Food, Anyang Institute of Technology, Huanghe Road, Anyang 455000, China; 3 School of Food Science and Engineering, Qilu University of Technology (Shandong Academy of Sciences), Jinan 250353, China; 4 Postdoctoral Workstation, Henan Yalinjie Biological Technology Co., LTD, Anyang 455000, China

**Keywords:** star anise, essential oil composition, hydroxypropyl-β-cyclodextrin, encapsulation, molecular modeling, volatilization stability, antibacterial properties

## Abstract

Star anise essential oil (SAEO) is a plant essential oil with good antibacterial activity, but its applications are limited due to its high volatility, strong smell, and unstable physical and chemical properties. The effect of selective encapsulation of SAEO by hydroxypropyl-β-cyclodextrin (HPCD) on its compositions, volatility stability and antibacterial activity was investigated. The GC-MS results indicated that the compositions reduced and content of the compositions of SAEO changed after encapsulation. Most of the components in SAEO were successfully encapsulated by HPCD, which can be supported by data from FTIR and ^1^H NMR. According to the molecular modeling results, the three guest molecules (*trans*-anethole, estragole and *trans*-foeniculin) were all docked in the cavity of HPCD on the isoallyl (or allyl) side. The volatile stability of SAEO before and after encapsulation was evaluated by electronic nose, and the results confirmed that encapsulation significantly reduced the irritating smell of SAEO and makes the clathrate have a sustained release effect. Furthermore, in the antibacterial test, the selective encapsulation of HPCD improved the inhibition effect of SAEO on *Rhizopus stolonoifer*, *Saccharomyces cerevisiae*, and *E. coli* and its antibacterial stability in 24 h.

## 1. Introduction

Star anise (*Illicium verum*, Hooker f.) belongs to the Magnoliaceae or Magnolia family. Its fruit is one of the most important spices, and it is indigenous to Southeastern China. Moreover, it has many beneficial functions due to its antioxidant [1], antibacterial, liver cancer preventative [2], and insecticidal characteristics [3]. Star anise essential oil (SAEO), which is extracted from star anise fruits, accounts for 3–3.5 wt.% of fresh fruit and exceeds 8 wt.% of dried fruit by steam distillation [4]. SAEO is widely used in food and medicine because of its good biological activity [5]. It is extensively used in baked goods, confections, and alcoholic and soft drinks [6]. In addition, it can alleviate inflammatory responses [7] and is a common flavor in medicinal tea, cough mixtures, and pastilles.

In recent years, the spoilage microorganisms in food have become resistant to synthetic antimicrobial agents, which has aroused researcher’s interest in the study of natural antimicrobial agents. Natural ingredients in many plants can kill or inhibit the growth of harmful microorganisms in food. There is an increased interest focusing on essential oils as alternative agents for the control of food corruption and harmful microbial reproduction [8,9,10]. A number of reports have demonstrated the antibacterial properties of the SAEO [11,12,13]. The antibacterial activities of SAEO are related to its bioactive volatile components. The main components obtained by steam distillation, specifically *trans*-anethole, estragole and limonene, account for about 96% of the total components [14].

Although the antibacterial properties of SAEO have been the target of much attention, the applications of SAEO are limited due to its high volatility, strong smell, and unstable physical and chemical properties [15,16,17]. The encapsulation techniques based on cyclodextrins (CDs) can solve this problem [18]. CDs and its derivatives can encapsulate essential oils and volatile compounds to stabilize their properties, extend their storage capabilities, mask the irritating smell [19], afford slow release properties [20,21] and enhance the antioxidant activity [22,23]. In addition, the main components of SAEO are volatile, and encapsulation can regulate their release, because the control of the release rate is the key of prolonging the antimicrobial characteristics of SAEO. Encapsulation of essential oil and its active constituents has been reported by several researchers. In 2014, the inclusion complexes of thymol and thyme essential oil with β-CD were successfully prepared to validate that the complexes exhibited good antibacterial activity [24]. Furthermore, several researchers successfully encapsulated black pepper essential oil in hydroxypropyl-β-cyclodextrin (HPCD). The results exhibited a slight decrease in the oxidation resistance of the inclusion complexes, while the antibacterial activity of black pepper oil improved by four-fold against both *S. aureus* and *E. coli* due to a likely synergistic effect between the wall material and essential oil [25].

CDs are cyclic oligosaccharides, occurring in nature by bacterial degradation of starch. In these natural processes, CDs have six, seven, or eight glucose units linked by α-(1,4)-glycosidic bonds, being named α-, β-, and γ-cyclodextrin, respectively. CDs have a cyclic molecular space structure, and exhibit special spatial properties, specifically a hydrophobic endothecium and hydrophilic exothecium, in aqueous solutions. This property allows CDs to form an inclusion complex with appropriately-sized lipid-soluble molecules [26], thereby improving the water solubility, bioavailability, or stability of the encapsulated molecules. The sustained release of the inclusion compound reduces the volatile loss of the essential oil [27]. HPCD is a derivative of β-CD and is introduced to the hydroxypropyl groups in the molecular structure of β-CD to enhance its water solubility and toxicological properties. SAEO contains a variety of volatile components, such as terpenes, terpenoids and phenol-derived aromatic components, and the compositions are vital for their antimicrobial activities. Moreover, the encapsulation ability of HPCD is different toward a variety of components, which may affect the biological activity of SAEO. However, to our knowledge, few reports have focused on molecular modeling of selective encapsulation of SAEO by HPCD and changes of composition, volatility stability, and antibacterial activity of SAEO after HPCD encapsulation.

The present study investigates the effect of selective encapsulation of SAEO by HPCD on its components, volatility stability, and antibacterial activity. The component change after the SAEO encapsulated in HPCD was determined through a solvent extraction and GC-MS method, and the inclusion complex was characterized by FTIR and ^1^H NMR. According to the principle of lowest energy, the structure of the host and guest after encapsulation was simulated by Hyperchem 8.0 issued by Hypercube, Inc. (Gainesville, FL, USA). The volatility stability of SAEO before and after encapsulation was described by electronic nose for the first time. Furthermore, the effect of HPCD’s selective encapsulation of SAEO on its antibacterial properties was evaluated via the restraint of its in vitro microbial growth (i.e., *Rhizopus stolonoifer*, *Saccharomyces cerevisiae*, and *E. coli*).

## 2. Results and Discussion

### 2.1. GC-MS Analyses

The GC-MS analysis techniques are often employed to identify the components of essential oils [28]. The identification of a single compound is based on the retention time and mass spectrometry of the sample. The GC-MS analysis of the SAEO before and after encapsulation is presented in Table 1. It can be seen from the Table 1 that the compositions and relative contents of SAEO after encapsulating changed, which may affect its antibacterial properties. Thirteen of SAEO main components were identified by GC-MS, which represent 97.09% ± 2.35% of all the components in SAEO (Table 1). The major components were identified as *trans*-anethole (91.38% ± 0.98%), estragole (2.55% ± 0.41%), and *trans*-foeniculin (2.15% ± 0.65%). This result is similar to that of Aly et al. [14], though the content of the compounds is slightly different.

During the extraction of the encapsulation complex, 12 components were identified, which constituted for 99.00% ± 1.93% of the gross components. Among these components, the relative contents were dominated by *trans*-anethole (95.36% ± 1.09%), estragole (2.23% ± 0.33%), and *trans*-foeniculin (0.91% ± 0.40%). Furthermore, the main components of the SAEO were the same before and after encapsulation. One of components was not identified in the extract due to its low content. In addition, the relative contents of the other SAEO components were all declined observably, except for *trans*-anethole and *cis*-anethole, which indicates that the *trans*-anethole and *cis*-anethole are more likely to be encapsulated in HPCD. These changes may be due to the difference in structure and polarity of the different constituent molecules in the SAEO and selective encapsulation of HPCD. The encapsulation effect of the HPCD on the guest molecules may be closely related to the structure of the guest molecules. According to Table 1, the structures of the three guest molecules with relatively high contents were similar, i.e., an isoallyl (or allyl) is connected to the benzene ring and an ether bond is connected with the benzene on the side of the *para*-position. However, the group connected to the structure mentioned above in *trans*-foeniculin was much more complex than that in *trans*-anethole and estragole. Consequently, it was more difficult to enter the HPCD cavity and form clathrate, which may have resulted in the bad encapsulate effect observed on the HPCD of *trans*-foeniculin.

### 2.2. FT-IR Spectra Studies

**Figure 1:**
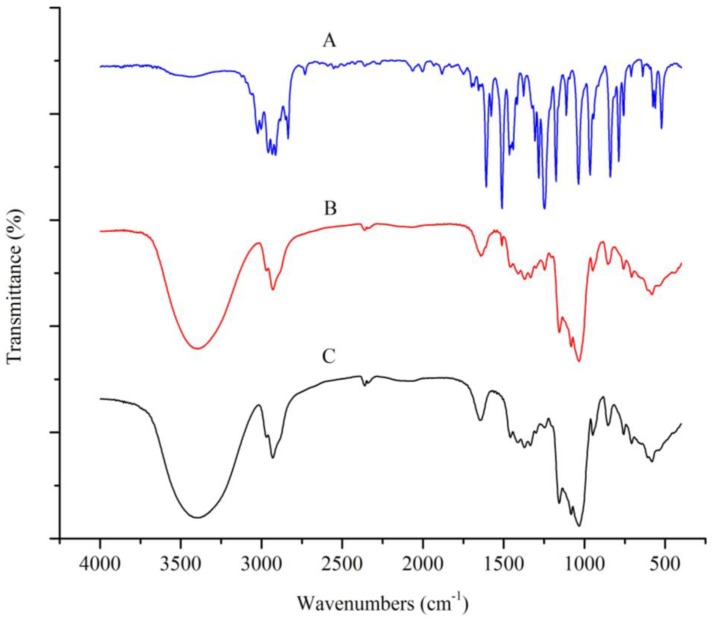
IR spectra of SAEO (**A**), HPCD (**C**) and their inclusion complex (**B**).

The IR spectra can be used to identify and analyze the compounds by the vibrational and rotational transitions of the molecules. Changes in the IR absorption peaks of the host and guest molecules can provide important information regarding the formation of the encapsulation complexes. Figure 1 presents the infrared spectra of SAEO, the HPCD, and their encapsulation complex.

According to the IR spectra of the SAEO, very strong absorption peaks were observed at 3020 cm^−1^ and 3008 cm^−1^, which represent the C-H stretching vibration peaks of the benzene ring and the C=C, respectively. The four sharp absorption peaks between 1609 cm^−1^ to 1447 cm^−1^ (1609, 1507, 1464, and 1447 cm^−1^) is defined by the stretching vibration peaks of the benzene ring framework. The stretching vibration peaks of C-O-C were represented by the peaks at 1248, 1175, and 1038 cm^−1^. The C-H bending vibration peaks of the substituent aromatics are represented by the peaks at 842 cm^−1^ and 790 cm^−1^. The relative content of *trans*-anethole in the SAEO was calculated to be 91.38% ± 0.98%. Therefore, the IR spectrum of the SAEO was very similar to the spectrum of *trans*-anethole [21]. The above information indicated that the molecules of the main constituents in the SAEO contained C=C bonds and aromatic ether bonds. This inference was consistent with the above-mentioned GC-MS analytical results.

Figure 1 reveals that the IR spectrum of the complex is very similar to that of HPCD. In addition, some characteristic absorption peaks (3020, 1609, and 790 cm^−1^) of SAEO in the IR spectra of the inclusion complex were not identified. These bands are masked due to overlapping with the more intense bands of HPCD. However, the absorption peaks at 1507 and 1248 cm^−1^ exhibit a decrease in the intensity, whereas that at 842 cm^−1^ exhibits a slight shift due to the stretching vibration of C-O-C and the bending vibration of C-H of the *para*-substituted benzene [29,30]. This could be explained by the stretching vibrations of the benzene framework and aromatic ether bond in the SAEO molecules being restricted following the formation of the inclusion and the low guest quantity in the inclusion complexes. The above information shows that the benzene ring and the aromatic ether bond in the SAEO molecules entered the cavity of the HPCD, and indirectly validates that some SAEO components were successfully encapsulated by the HPCD.

### 2.3. ^1^H NMR Spectra Analyses

^1^H NMR can provide valuable information about the spatial position of guest molecules in the cyclodextrin cavity, as well as the formation and dissociation encapsulation conditions in the solvent. H3 and H5 are atoms in the inner wall of the cyclodextrin cavity. When the guest molecules enter the cavity, the chemical shift of the H3 and H5 atoms in the cyclodextrin cavity changed due to the interaction between the guest molecule and the hydrophobic cavity of the cyclodextrins [31].

**Figure 2:**
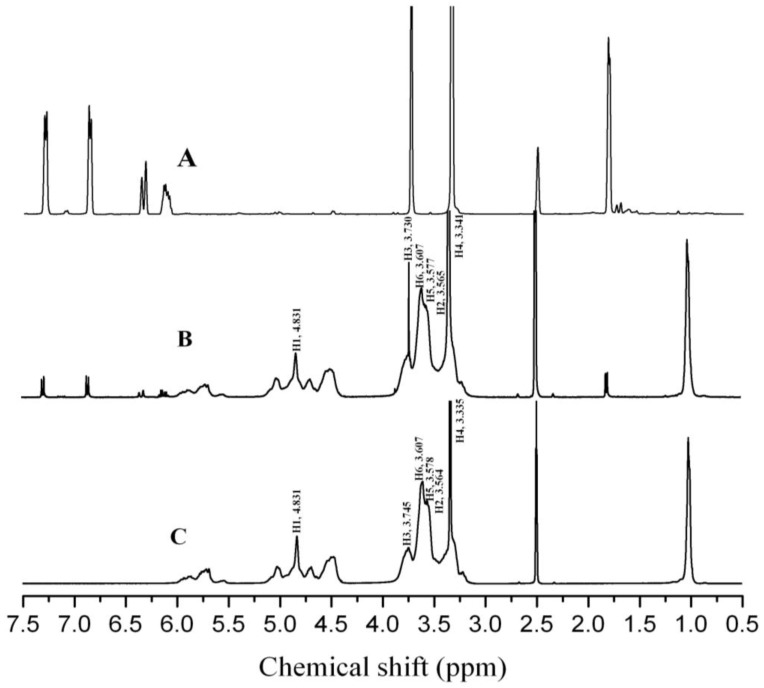
^1^H NMR spectra of SAEO (**A**), the inclusion complex (**B**), and HPCD (**C**).

Figure 2 presents the ^1^H NMR spectra of SAEO, the inclusion complex, and the HPCD. The ^1^H NMR diagram of SAEO exhibited obvious proton peaks at 7.3 and 6.8 ppm, which indicates that the molecular structure of the compositions in SAEO contains a benzene ring. The proton peaks near 6.3 and 6.1 ppm indicate the presence of a double bond in the molecular structure of the chemical composition of SAEO. The proton peaks near 3.3 and 3.7 ppm symbolize that the SAEO molecular composition contains a C-O-C structure. The proton peak near 1.8 ppm is a characteristic peak of H on the methyl or methylene structure linked to the double bond. These results were well in agreement with the analytical results of the GC-MS analysis. The ^1^H NMR diagram of the encapsulation complex exhibited the presence of both HPCD and SAEO proton peaks. Figure 2B,C presents the chemical shift changes in the protons of the D-glucopyranose units in the HPCD molecules. No changes were observed in the H1 atoms in the mid-structure and H6 atoms at the outermost side of the HPCD cavity, whereas the H2 and H4 atoms outside the cavity shifted slightly towards the low field. The H3 atoms near the wider edge in the cavity visibly shifted towards the high field, and the H5 atoms in the depth of the cavity also exhibited a high field shift. Based on the ^1^H NMR spectra analysis of the SAEO and the inclusion complex, the benzene ring structure and the double bond entered the cavity of HPCD. The stronger shielding effect of the benzene ring and the double bond resulted in an obvious increase in the electron cloud density around the H3 and H5 atoms and a decrease in the chemical shift. As a result, the H3 and H5 atoms shifted towards the high field. These changes indicate that the inclusion complex has been successfully formed.

### 2.4. Molecular Modeling Studies

**Figure 3:**
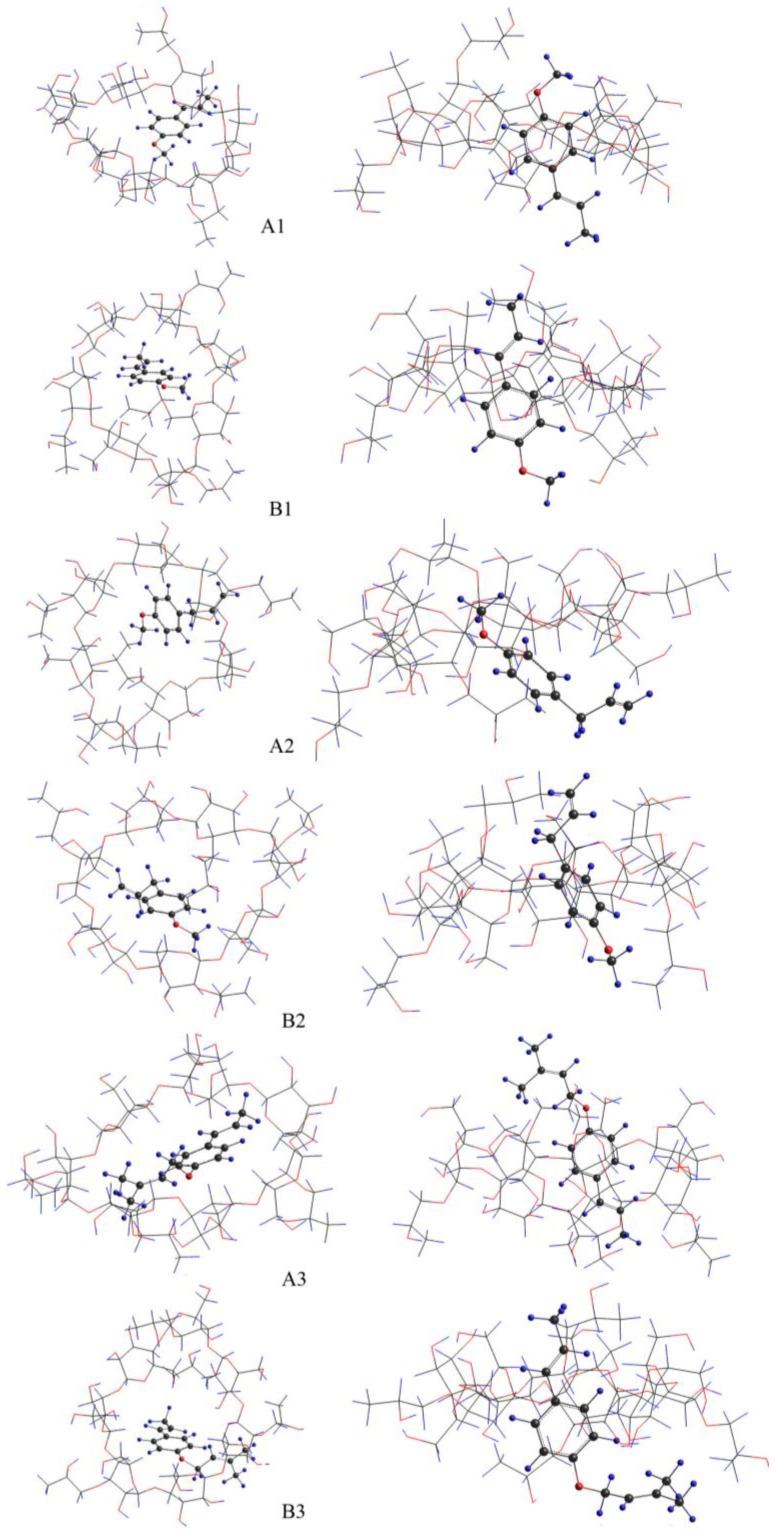
Energy-minimized modes obtained by the PM3 calculations for the complex of *trans*-anethole (**A1** and **B1**), estragole (**A2** and **B2**), and *trans*-foeniculin (**A3** and **B3**) with HPCD. Left, top view; right, side view.

At present, molecular modeling based on molecular mechanics is been widely applied to characterize the three-dimensional structure of inclusion complexes with cyclodextrins [32]. The above results indicate that the main components of SAEO in the encapsulation compound were *trans*-anethole, estragole, and *trans*-foeniculin, of which the possible molecular models and corresponding three-dimensional encapsulation structures were generated according to the PM3 method of Hyperchem 8.0. The models of the three types of inclusion complexes obtained by the methods in Section 3.6 are presented in Figure 3. The ΔE of model A (1.595 kcal/mol) of the *trans*-anethole molecule was positive and was significantly higher than that of model B (−9.067 kcal/mol) given that the encapsulating process reduced the energy of the CDs. As a result, it is unreasonable to generate model A of the *trans*-anethole molecule. The ΔE of model B (−7.881 kcal/mol) was slightly lower than that of model A (−7.323 kcal/mol) of the estragole molecule. The ΔE of model B (−14.583 kcal/mol) was significantly lower than that of model A (−1.973 kcal/mol) of the *trans*-foeniculin. Therefore, model B of the three guest molecules exhibited the most reasonable inclusion complex structure. Figure 3(B1–B3) indicates that all three guest molecules were inserted into the cavity of HPCD of the isoallyl (or allyl) side. Moreover, the isoallyl (or allyl) group was deeply inserted, and the benzene ring was observed near the wide edge of the HPCD, whereas the ether bonds were exposed outside the HPCD cavity. The formation of the inclusion complex was largely a result of the addition of the benzene ring of the three molecules to the hydrophobic HPCD cavity by hydrogen bonding, which is consistent with the analysis results of FT-IR and ^1^H NMR.

### 2.5. Volatile Stability

**Figure 4:**
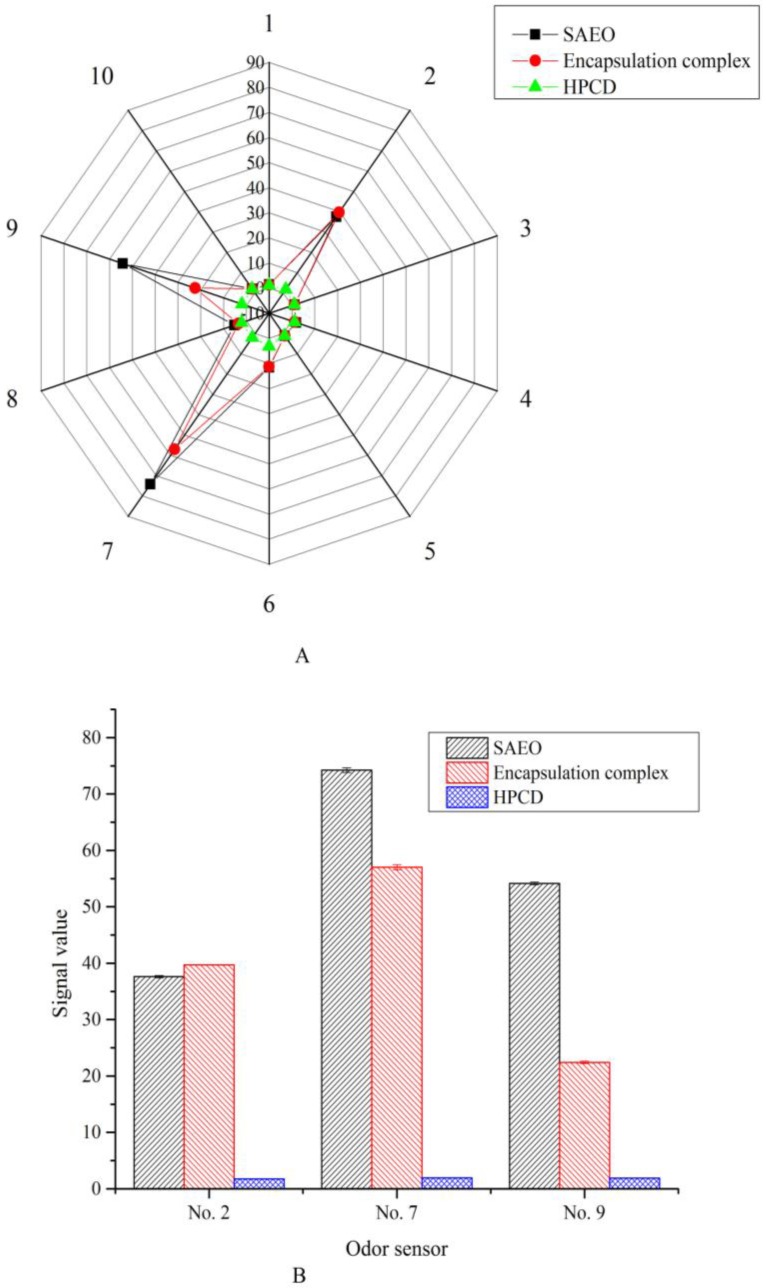
Analysis results of volatile stability of SAEO, the encapsulation complex and HPCD: (**A**) radar-plot detected using 10 sensors; (**B**) bar-plot based on responses of relatively sensitive sensors No. 2, 7 and 9.

SAEO contains a large number of volatile components with a strong smell, and that volatile losses may affect its antibacterial activity, and the pungent smell will limit its application in food preservation. This volatile loss can be effectively reduced after encapsulating SAEO to HPCD, while masking this pungent smell without diminishing its biological activity [25]. Electronic nose technology is an artificial intelligence technology that simulates the human sense of smell, and is widely used in quality analysis and classification of essential oils [33]. The results of the comparison of volatile stability of SAEO, inclusion complex, and HPCD are shown in Figure 4. Figure 4A shows that the volatile components in the emulsion of SAEO and inclusion complex were sensitive to sensors no. 2, no. 7, and no. 9. The sensors no. 2, no. 7, and no. 9 indicate “broad range”, “sulfur-organic” and “sulf-chlor”, respectively. The electronic nose describes the smell characteristics of SAEO as “sulfur-organic” and “sulf-chlor”, which may be related to its strong volatile irritation. It is also shows from Figure 4A that there is a difference in the shape of the smell radar map of the SAEO and clathrate. This result corroborates the “changes in the composition and content of SAEO after the HPCD encapsulation” analyzed by the GC-MS analysis. The signal values detected by no. 7 and no. 9 sensors in Figure 4B shows that the “sulfur-organic” and “sulf-chlor” smell of the emulsion of SAEO was significantly stronger than that of the encapsulation solution. This indicates that the embedding of HPCD masks the partial irritating smell of SAEO, which makes the encapsulation compound have a sustained release effect.

### 2.6. In Vitro Antimicrobial Activity

**Figure 5:**
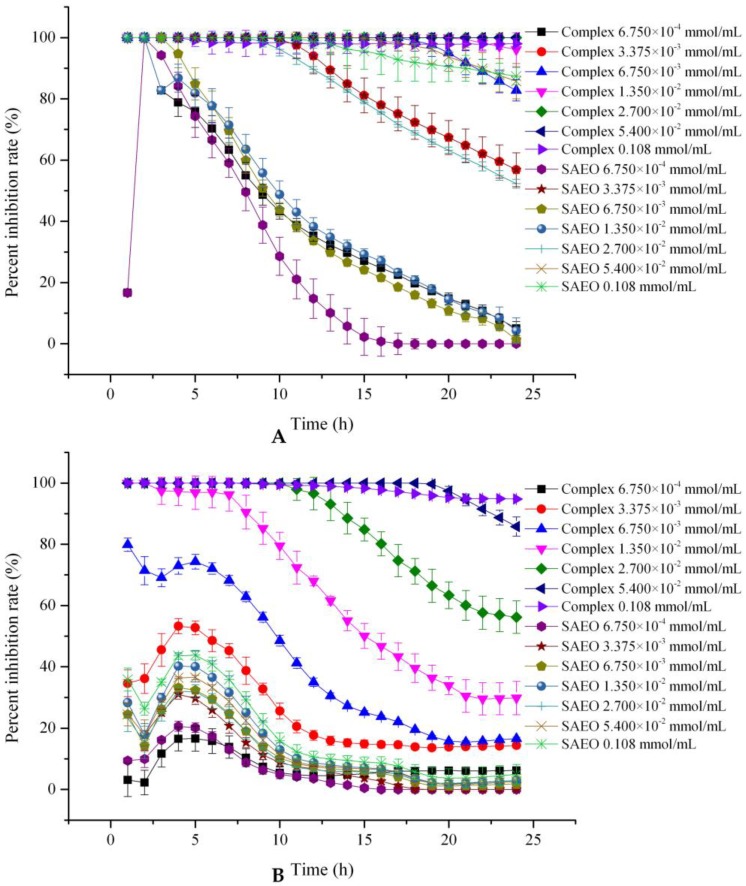

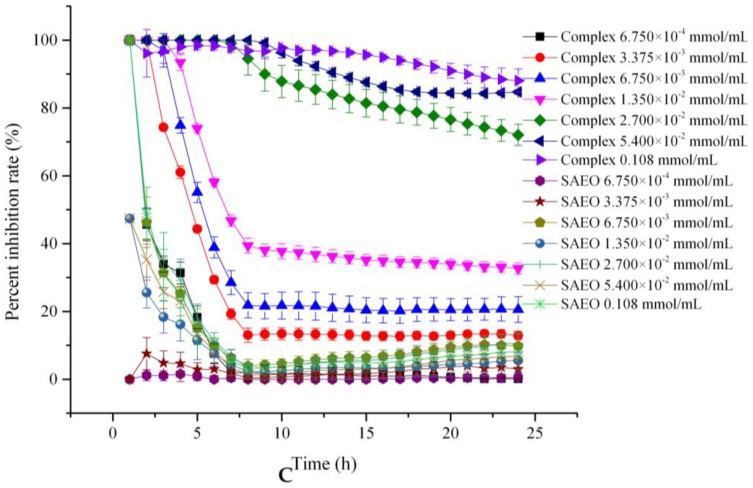
The inhibition effect of SAEO and the inclusion complex with HPCD on *Rhizopus stolonoifer* (**A**); *Saccharomyces cerevisiae* (**B**) and *E. coli* (**C**).

According to the previous literature, SAEO is beneficial in its ability to inhibit microorganism growth [34]. However, its instability and strong volatility challenges its widespread application in food preservation. The present study described the capsulation method of SAEO to the HPCD cavity, of which the restraining effects on *Rhizopus stolonoifer*, *Saccharomyces cerevisiae*, and *E. coli* before and after encapsulation were compared. According to Table 2 and Figure 5, SAEO and the inclusion complex had inhibitory effects on *Rhizopus stolonoifer*, *Saccharomyces cerevisiae*, and *E. coli*, the inhibition effect against *Rhizopus stolonoifer* was better than that of *Saccharomyces cerevisiae* and *E. coli*, which is consistent with the reported results in the literature [12]. In addition, under the same concentration, the antibacterial effect of the clathrate was obviously better than that of the free SAEO. It is possible that the relative content of *trans*-anethole, the main antibacterial component of encapsulated SAEO, increased (from 91.38% to 95.36%) due to the encapsulation selectivity of HPCD. Moreover, the encapsulation by HPCD improved the water solubility of SAEO so that the antibacterial components in SAEO can more easily penetrate the cell membrane of microorganism, thereby exerting the inhibitory effect. Figure 5 also shows that the antibacterial stability of the inclusion complex was better than that of the free SAEO in 24 h, especially at two concentrations of 5.400 × 10^−2^ mmol/mL and 0.108 mmol/mL, which may be due to the encapsulation slowing the release of SAEO.

## 3. Materials and Methods

### 3.1. Materials

Star anise was purchased from Guangxi Rongxian Guoyao Agricultural Products Co., Ltd. (Yulin, China) and underwent the hydrodistillation method to generate SAEO and was dried with anhydrous sodium sulfate. HPCD (purity > 99%, average Mw = 1380) was purchased from Sigma-Aldrich Shanghai Trading Co. Ltd. (Shanghai, China). *Rhizopus stolonoifer* (separated from baked food), *Saccharomyces cerevisiae*, and *E. coli* were provided by the microbial laboratory in the Anyang Institute of Technology. Sabouraud dextrose broth (SDB) and tryptic soy broth (TSB) were purchased from Qingdao High Tech Industrial Park Hopebio Technology Co., Ltd. (Qingdao, China). Other reagents were of analytical grade. The water used was double-distilled and deionized.

### 3.2. Preparation of the Inclusion Complex of SAEO with HPCD

The SAEO inclusion complexes were prepared by the freeze-drying method according to the published procedure [35]. SAEO was added to 25 mL HPCD aqueous solution with the mole ratio of 1:1. The mixture was ultrasonically treated and magnetically stirred for 96 h at 30 °C in the dark. The encapsulation solution was filtered through 0.45 μm filters to eliminate any undissolved compounds after the complexation reaction. The filtrates were lyophilized at −60 °C and 100 Pa in a Millrock Technology BT85 freeze dryer (Millrock Technology, Inc., Kingston, NY*,* USA).

### 3.3. GC-MS Analyses

SAEO was accurately weighed and dissolved in n-hexane to prepare sample solution A at a concentration of 0.003 mg/mL. Ten milligrams of SAEO/HPCD encapsulation compound was accurately weighed and dissolved in 5 mL deionized water, after which 10 mL n-hexane was added. The sample underwent ultrasonic extraction for 10 min to prepare sample solution B. The constituents of SAEO before (sample solution A) [36] and after (sample solution B) encapsulation were analyzed by GC-MS (Agilent 7890A-5975C, Santa Clara, CA, USA).

The GC conditions are presented as follows: J and W 122-5532 quartz capillary column (30 m × 250 μm × 0.25 μm); an inlet temperature of 250 °C; oven temperature was maintained at 60 °C for 1 min, after which it was elevated by 2 °C/min to 130 °C and subsequently elevated by 5 °C/min to 240 °C, at which the temperature was maintained for 1 min; a split ratio of 15:1; carrier gas (99.999% He); a velocity of 0.95 mL/min; and an injection volume of 1 μL. The MS conditions are presented as follows: electron impact (EI) ion source; an ion source temperature of 230 °C; an MS quadrupole temperature of 150 °C; and a mass scan range of 30–500 amu.

### 3.4. FT-IR Spectra

Approximately 1 mg HPCD and 1 mg of the inclusion complex samples were placed in an agate mortar with about 100 mg dry potassium bromide to form a fine powder, respectively. The sample was then mixed well, loaded into a mold, pressed into tablets, and placed in the Bruker Tensor II FT-IR spectrometer (Karlsruhe, Germany) for testing, respectively. The SAEO tablet sample was prepared by adding a drop of SAEO to the potassium bromide tablet.

### 3.5. ^1^H NMR Spectra

The ^1^H NMR spectra were recorded at 25 °C with a Bruker AM-400 NMR spectrometer (Karlsruhe, Germany) at 500 MHz. SAEO, the encapsulation complex, and HPCD were dissolved in the DMSO solution, placed in NMR tubes with 5 mm inner diameters, and respectively tested.

### 3.6. Molecular Modeling

According to the above-mentioned test method in Section 3.3, the main constituents in SAEO before and after encapsulation were *trans*-anethole, estragole, and *trans*-foeniculin. *Trans*-anethole, estragole, *trans*-foeniculin, and HPCD were molecularly simulated using Hyperchem 8.0 issued by Hypercube, Inc. (Gainesville, FL, USA). The structures of *trans*-anethole, estragole, *trans*-foeniculin and HPCD were first constructed by means of Hyperchem 8.0 and then optimized by the PM3 method. The ether linkage side or isoallyl (or allyl) of the guest molecules were then inserted into the cavity of HPCD from the wide edge to the structure model A or model B, respectively. Both modes were minimized with the conjugate gradient optimizer until a root mean square (RMS) value of 0.01 kcal/(mol Å) was obtained [37]. The ΔE of the minimum energy mode was calculated on the Equation (1):

(1) $$\Delta E=E_{complex}-(E_{host}+E_{guest})$$

where *E_host_*, *E_guest_*, and *E_complex_* (kcal/mol) represent the calculated energy of the HPCD, the main components’ molecules in the SAEO, and the encapsulation molecular complex, respectively.

### 3.7. Volatile Stability

The ratio of the resistance (G/G0) of the volatile gas in the sample to the blank was obtained by the 10 gas sensors of the PEN3 electronic nose (Schwerin, Germany), in order to describe the volatile stability of the sample. That is, the smaller the ratio, the better the volatility stability. Twelve milligrams of SAEO and the encapsulation complex containing the same amount of SAEO were added to the sample bottle with 5 mL deionized water, respectively. Their volatility stability data were detected by 10 gas sensors of PEN3 electronic nose after standing for 30 min at room temperature. The same method was used to detect the volatility stability of HPCD. All the test samples were performed in triplicate.

### 3.8. In Vitro Antimicrobial Activity

Antimicrobial activity and the MICs analyses of free and encapsulated SAEO were performed against *Rhizopus stolonoifer*, *Saccharomyces cerevisiae*, and *E. coli* (provided by the microbial laboratory in the Anyang Institute of Technology) in 96-well microtiter plates by several researchers with minor modification [25,38,39]. These strains representing typical spoilage organisms commonly exist in food products. *Rhizopus stolonoifer* and *Saccharomyces cerevisiae* were cultured on SDB at 28 °C, and *E. coli* was cultured on TSB at 37 °C. The suspensions with strains (*Rhizopus stolonoifer*, *Saccharomyces cerevisiae*, and *E. coli*) concentration of approximately 10^5^ CFU/mL were prepared and 1 mL of the bacterial suspension was added to 150 mL of liquid medium. Three-hundred microliters of inoculated culture medium was added to each well of the microplate. Furthermore, 0.000, 6.750 × 10^−4^, 3.375 × 10^−3^, 6.750 × 10^−3^, 1.350 × 10^−2^, 2.700 × 10^−2^, 5.400 × 10^−2^, and 0.108 mmol/mL SAEO and the inclusion complex containing the same amount of SAEO were added to the inoculated culture medium, respectively. That is, the concentrations of SAEO in the inoculated liquid medium containing inclusion complex are also 0.000, 6.750 × 10^−4^, 3.375 × 10^−3^, 6.750 × 10^−3^, 1.350 × 10^−2^, 2.700 × 10^−2^, 5.400 × 10^−2^, and 0.108 mmol/mL, respectively. The culture medium solution was mixed by a vortex oscillator to ensure good distribution. Incubations of *Rhizopus stolonoifer* and *Saccharomyces cerevisiae* were carried out in a dark room at 28 °C for 24 h, and the incubation of *E. coli* was performed at 37 °C for 24 h. The OD value of liquid culture medium containing inoculums was then measured at 600 nm once every 1 h by a Bioscreen C automatic analyzer for microbial growth curves (Turku, Finland), respectively. The inhibition rate was calculated on the Equation (2). Each test was performed in triplicate:

(2) $$\mathrm{Inhibition}\;\mathrm{rate}(\%)=\frac{\Delta OD_{C}-\Delta OD_{S}}{\Delta OD_{C}}\times 100\%$$

where ∆*OD_C_* and ∆*OD_S_* are the value change of the control sample and the post-treatment samples with SAEO or the SAEO/HPCD inclusion complex at *OD* of 600 nm after a certain period of incubation time, respectively.

The MICs of free and encapsulated SAEO were determined using a microdilution assay [25]. The antimicrobial inclusion complexes were added to the microtiter plates as aqueous suspensions, while the SAEO was added as aqueous microemulsions. The concentration of inclusion complexes added to the test wells ranged from 62.5 to 1000 mg/mL (equivalent to 1.25–20 mg/mL of SAEO concentration based on the entrapment efficiency), while the concentration of free SAEO ranged from 1.25 to 20 mg/mL.

Negative control wells were prepared with sterile culture medium containing tested samples (free and encapsulated SAEO). Positive control wells were prepared with microbial suspension inoculating in culture medium. The microplates were incubated at 37 °C or 28 °C for 24 h and the turbidity was determined at 600 nm. The MICs of free and encapsulated SAEO were recorded as the lowest concentration where no visible growth (≤0.05 changed in *OD*_600_) was observed in the wells after 24 h of incubation.

## 4. Conclusions

The present study investigated the characteristics of SAEO after encapsulation in HPCD. The results of the FT-IR and ^1^H NMR spectra confirmed that SAEO was successfully encapsulated in the HPCD cavity. Hydrophobic SAEO became a water-soluble encapsulation complex. However, the compositions of SAEO and its relative content were different from those before encapsulation. In addition to *cis*-anethole and *trans*-anethole, the relative content of most components decreased. The components that contained an ether bond were more easily encapsulated into the HPCD cavity as compared to the other components, which may result in changes in the antimicrobial properties of SAEO. The results of the molecular modeling indicated that the embedded modes of the three main components were inserted into the cavity of HPCD on the isoallyl (or allyl) side. In addition, the components with higher contents formed complexes with HPCD more easily. The volatile stability of SAEO after encapsulation was evaluated by electronic nose, and the data showed that the “sulfur-organic” and “sulf-chlor” smell of the encapsulation solution was significantly lower than the SAEO and water mixture due to the embedding effect of the HPCD. This indicated that encapsulation significantly reduced the irritating smell of SAEO and makes the clathrate have a sustained release effect. The results of the antibacterial test indicated that the inhibitory effect of SAEO on *Rhizopus stolonoifer*, *Saccharomyces cerevisiae*, and *E. coli* markedly increased following the formation of the encapsulation complex due to the improvement of water solubility of SAEO. Furthermore, the antibacterial stability of the inclusion complex in 24 h was generally superior to that of free SAEO on account of the encapsulated slow release effect.

## Figures and Tables

**Table 1:** Composition analysis results of SAEO before (A) and after (B) the encapsulation ^1^.

No.	Retention Time (Min)	Compound	Structure	Content in Sample A (%)	Content in Sample B (%)
1	18.656	Eucalyptol	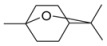	0.12 ± 0.06	0.07 ± 0.01
2	19.574	1-Methyl-5-(1-methylethenyl)-(R)-cyclo-hexene	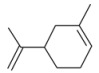	0.09 ± 0.01	0.04 ± 0.00
3	19.759	Estragole	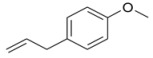	2.55 ± 0.41	2.23 ± 0.33
4	23.118	*cis*-Anethole	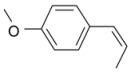	0.12 ± 0.05	0.16 ± 0.03
5	23.270	2-Hydroxy-4-methyl-benzaldehyde	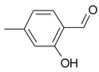	0.08 ± 0.01	- ^2^
6	25.243	*trans*-Anethole	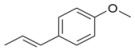	91.38 ± 0.98	95.36 ± 1.09
7	25.724	o-Allyloxytoluene	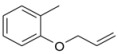	0.04 ± 0.01	0.01 ± 0.00
8	33.042	α-Farnesene	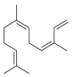	0.08 ± 0.02	0.01 ± 0.01
9	33.240	Caryophyllene	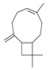	0.14 ± 0.05	0.07 ± 0.02
10	34.251	*trans*-α-Bergamote-ne	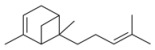	0.18 ± 0.07	0.11 ± 0.03
11	35.693	Salicylic acid	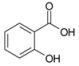	0.09 ± 0.02	0.02 ± 0.01
12	44.487	*trans*-Foeniculin	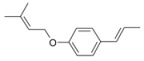	2.15 ± 0.65	0.91 ± 0.40
13	54.351	*trans*-Chalcone	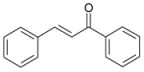	0.08 ± 0.01	0.01 ± 0.00
Total				97.09 ± 2.35	99.00 ± 1.93

^1^ All tests were performed in triplicate and the results are expressed as the mean ± standard deviation; ^2^ “-” means not detected.

**Table 2:** The minimum inhibitory concentrations (MICs) of SAEO and the inclusion complex on *Rhizopus stolonoifer*, *Saccharomyces cerevisiae*, and *E. coli.*

Antibacterial Agents	MICs (mg/mL)		
	*Rhizopus stolonoifer*	*Saccharomyces cerevisiae*	*E. coli*
SAEO	20	>20 ^1^	>20
Iclusion complex ^2^	2.5	20	20

^1^ Values preceded by a greater than symbol (>) means that tested concentrations were not sufficient to determine the MIC values; ^2^ Values are based on the actual concentrations of SAEO encapsulated in the HPCD (calculated from encapsulation efficiency).
